# Aristaless Related Homeobox Gene, *Arx*, Is Implicated in Mouse Fetal Leydig Cell Differentiation Possibly through Expressing in the Progenitor Cells

**DOI:** 10.1371/journal.pone.0068050

**Published:** 2013-06-28

**Authors:** Kanako Miyabayashi, Yuko Katoh-Fukui, Hidesato Ogawa, Takashi Baba, Yuichi Shima, Noriyuki Sugiyama, Kunio Kitamura, Ken-ichirou Morohashi

**Affiliations:** 1 Department of Molecular Biology, Graduate School of Medical Sciences, Kyushu University, Fukuoka, Japan; 2 Department of Molecular Endocrinology, National Research Institute for Child Health and Development, Tokyo, Japan; 3 Advanced ICT Research Institute Kobe, National Institute of Information and Communications Technology, Kobe, Japan; 4 Department of Anatomy and Developmental Biology, Graduate School of Medical Science, Kyoto Prefecture University of Medicine, Kyoto, Japan; 5 Department of Mental Retardation and Birth Defect Research, National Institute of Neuroscience, National Center of Neurology and Psychiatry, Kodaira, Tokyo, Japan; Clermont Université, France

## Abstract

Development of the testis begins with the expression of the *SRY* gene in pre-Sertoli cells. Soon after, testis cords containing Sertoli and germ cells are formed and fetal Leydig cells subsequently develop in the interstitial space. Studies using knockout mice have indicated that multiple genes encoding growth factors and transcription factors are implicated in fetal Leydig cell differentiation. Previously, we demonstrated that the *Arx* gene is implicated in this process. However, how ARX regulates Leydig cell differentiation remained unknown. In this study, we examined *Arx* KO testes and revealed that fetal Leydig cell numbers largely decrease throughout the fetal life. Since our study shows that fetal Leydig cells rarely proliferate, this decrease in the KO testes is thought to be due to defects of fetal Leydig progenitor cells. In sexually indifferent fetal gonads of wild type, ARX was expressed in the coelomic epithelial cells and cells underneath the epithelium as well as cells at the gonad-mesonephros border, both of which have been described to contain progenitors of fetal Leydig cells. After testis differentiation, ARX was expressed in a large population of the interstitial cells but not in fetal Leydig cells, raising the possibility that ARX-positive cells contain fetal Leydig progenitor cells. When examining marker gene expression, we observed cells as if they were differentiating into fetal Leydig cells from the progenitor cells. Based on these results, we propose that ARX acts as a positive factor for differentiation of fetal Leydig cells through functioning at the progenitor stage.

## Introduction

In mammals, gonadal sex is determined by the presence or absence of the sex-determining gene, *SRY,* whose expression acts as a cue for differentiation from a sexually indifferent gonad into the testis [1]–[4]. The fetal testis is composed of germ cells and several types of somatic cells such as Sertoli and Leydig cells. Germ and Sertoli cells are distributed within testis cords, while steroidogenic Leydig cells and as yet uncharacterized cells remain in interstitial space. Among these cells, *SRY* is expressed only in pre-Sertoli cells to determine their cell fate into Sertoli cells. Soon after the Sertoli cell differentiation, sex-dependent events such as differentiation of steroidogenic Leydig cells and suppression of mitotic division of male germ cells [5] are induced possibly through signals from Sertoli cells.

Gene knockout (KO) mouse studies have demonstrated that growth factors are involved in differentiation of fetal Leydig cells, which are responsible for androgen production in male fetuses. This differentiation was suppressed in the fetal testes of *Dhh* (Desert hedgehog) KO mice [6], [7]. Consistent with the phenotype, activation of Dhh signaling enhanced differentiation of fetal Leydig (steroidogenic) cells in the ovary [8]. Similarly, suppression of fetal Leydig cell differentiation occurred in the testes of *Pdgfrα* (platelet derived growth factor receptor α, which is normally expressed in interstitial cells) KO mice [9]. Moreover, when Notch signaling was activated in fetal testes by genetic manipulation, differentiation of fetal Leydig cells was suppressed [10]. In contrast, blocking of Notch signaling resulted in an increase of fetal Leydig cells [10]. Disruption of *Wnt4* (wingless-related MMTV integration site 4), of which expression is enriched in the developing fetal ovary [11], resulted in an ectopic appearance of fetal Leydig (steroidogenic) cells in the ovary. Taken together, it has been demonstrated that Dhh and Pdgf signalings positively regulate, while Notch and Wnt4 signalings negatively regulate fetal Leydig cell differentiation during gonadal development. In addition to these growth factors, involvement of transcription factors into fetal Leydig cell differentiation has been reported. When the *Pod1* gene (E-box binding transcription factor, capsulin/epicardin/nephgonadin/Tcf21) was disrupted, fetal Leydig cell differentiation was activated [12].

*Arx* (Aristaless related homeobox gene), an X-linked gene related to the *Drosophila aristaless*, is conserved among vertebrate species. *Arx* is expressed in the forebrain, floor plate, gonad, pancreas, olfactory system, and skeletal muscle of mouse fetuses [13]–[17]. Gene KO studies have revealed some crucial functions of *Arx* during differentiation of the tissues/cells above [14]–[17]. Our previous study indicated that differentiation of fetal Leydig cells is affected in the *Arx* KO testis [14]. Consistent with this, the seminal vesicle, whose development is regulated by androgen, was underdeveloped in the KO mice [14].

X-linked lissencephaly with ambiguous genitalia (XLAG) is a syndrome occurring in humans that is characterized by symptoms such as abnormalities in neural and reproductive systems [18], [19]. Considering the symptoms in the human patients and the X-linked gene locus responsible for the disease, *ARX* was proposed as one of the candidate genes and subsequently sequencing of patients’ DNA confirmed that *ARX* is responsible for XLAG [14].

In the present study, we examined the expression of *Arx* in developing gonads throughout the fetal stage and gonadal defects induced in *Arx* KO mice. As *Arx* may be expressed in fetal Leydig progenitor cells and fetal Leydig cell differentiation is affected at the progenitor stage in the *Arx* KO mice, we propose that ARX acts as a positive regulator for differentiation of fetal Leydig cells through expressing and functioning at the progenitor stage.

## Materials and Methods

### Mice

*Arx* KO mice (Arx-1 KO (03455)) [14] were provided by RIKEN BRC through the National Bio-Resource Project of the MEXT, Japan. *Arx* KO and *Wnt4* KO [11], [20] mice were maintained as closed colonies. ICR mice were purchased from Japan SLC, INC (Hamamatsu, Japan). All protocols for animal experiments were approved by the Institutional Animal Care and Use Committee of Kyushu University (Permit Number: A24-060-1). Mice were sacrificed after Sevoflurane anesthesia, and all efforts were made to minimize suffering.

### Immunohistochemistry, Immunofluorescence, HE Staining, and in situ Hybridization

Paraffin sections (5 µm) and cryosections (10 µm) were prepared from wild type and KO mouse fetuses fixed in 4% paraformaldehyde at 4°C overnight. Immunohistochemistry and immunofluorescence were performed as described previously [21]. The sections were boiled for 10 min in 10 mM sodium citrate to unmask antigen epitopes. 10 mM sodium citrate at pH 6.0 was used for immunostaining of ARX, AD4BP/SF-1 (Adrenal-4 Binding Protein [22], Steroidogenic Factor-1 [23], NR5A1 [24]), SOX9 (Sry-related HMG box containing protein), LHX9 (LIM homeobox 9) [25], BrdU (bromodeoxyuridine), active-type caspase 3 and MAFB (v-maf musculoaponeurotic fibrosarcoma oncogene family, protein B), while that at pH 2.0 was used for MIS (Müllerian inhibiting substance), WT-1 (suppressor gene for Wilms’ tumor), and DAX1 (NR0B1 [24] [26]). Rabbit antibodies against ARX (1∶500) [14], AD4BP/SF-1 (1∶2000) [27], 3β-HSD (3β-hydroxysteroid dehydrogenase) (1∶2000) [28], SOX9 (1∶2000) [29], WT1 (Santa Cruz Biotechnology, Inc., Dallas, TX, C-19, 1∶100) [30], [31], LAMININ (Sigma, St Louis, MO, 1∶1000), active-type caspase 3 (BD Bioscience, San Jose, California, BD Pharmingen™, 559565, 1∶50) and MAFB (Bethyl Laboratories, Inc., IHC-00351, 1∶1000), rat antibodies against AD4BP/SF-1 (1∶100) [32], [33], PECAM-1 (1∶1000) (BD Bioscience, San Jose, California, BD Pharmingen™, MEC13.3, 1∶1000), and LHX9 (1∶50) [29], a goat antibody against MIS (Santa Cruz Biotechnology, Inc., C-20, 1∶200), a mouse antibody against BrdU (Roche, Indianapolis, IN, 1∶100), and a guinea pig antibody against DAX1 (1∶2000) [26] were used. Biotinylated anti-rabbit, anti-goat, and anti-guinea pig antibodies (Jackson ImmunoResearch, West Grove, PA), Alexa Fluor® 488-labeled anti-rabbit antibody, Alexa Fluor® 555-labeled anti-rat antibody (Molecular Probes, Eugene, OR), and Cy3-labeled anti-rat and anti-mouse antibodies were used as the secondary antibodies. Antigen-antibody complexes were detected using Histofine kit (Nichirei, Tokyo, Japan) or directly by fluorescence. Nuclei were counterstained with DAPI (Sigma). We used Can Get Signal® Immunostain Solution B (TOYOBO Co. Ltd., Osaka, Japan) for signal enhancement of MAFB staining. Paraffin sections were stained with hematoxylin and eosin (HE). *In situ* hybridization was performed as described [34]. Digoxigenin labeled riboprobes (Roche Diagnostics, Mannheim, Germany) for *Dhh*, *Ptch1*, *Pdgfrα* (kindly provided by Dr. Kazuhiro Ikenaka and Dr. Hirohide Takebayashi), *Wnt4* (kindly provided by Dr. Andrew McMahon) [20], and *Fst* (Follistatin) (kindly provided by Dr. David C. Page) [35] were used.

### Western Blotting

Testes prepared from mouse fetuses at E (embryonic day) 11.5, E12.5, E14.5, and E18.5 were lysed with 50 mM Tris-HCl (pH 8.0), 50 mM NaCl, 1 mM EDTA (pH 8.0), and 1% SDS. Five µg of whole tissue lysates were subjected to SDS-PAGE followed by western blot analyses using antibodies against ARX (1∶2000) [14] and α−tubulin (TUBA) (SIGMA, T6199, 1∶1000) [36].

### Counting of Leydig and Sertoli Cells

Testes prepared from wild type and *Arx* KO mouse fetuses (n = 2 and 3, respectively) were sectioned, and 6 sections for each gonad were randomly selected and stained with antibodies against 3β-HSD and SOX9. Numbers of 3β-HSD-positive Leydig cells and SOX9-positive Sertoli cells were counted, and whole gonadal areas were measured.

### Measurement of Intratesticular Testosterone

Testes were prepared from wild type and *Arx* KO mouse fetuses (n = 3) at E18.5 and stored at −80°C until the assay. Concentration of intratesticular testosterone was determined by LC-MS/MS (ASKA Pharma Medical, Kanagawa, Japan).

### Cell Proliferation Assay

Pregnant ICR females were sacrificed 2 hours after intraperitoneal injection of BrdU (Sigma) (50 mg/kg body weight) at E12.5, 14.5, and 16.5 [37]. Three fetuses were used for each gestational day. Paraffin sections of the fetuses were double immunostained for BrdU and 3β-HSD or BrdU and SOX9. The numbers of BrdU and 3β-HSD double-positive cells, BrdU and SOX9 double-positive cells, and single-positive cells for 3β-HSD or SOX9 were counted with more than 5 sections for each gonad.

### Quantitative RT-PCR

Total RNA was prepared from gonads of wild type (n = 8) and *Arx* KO mice (n = 10) at E12.5 by using RNeasy Micro Kit (QIAGEN). cDNAs were synthesized from the RNA samples by using Superscript II reverse transcriptase (Life Technologies) and oligo (dT)_15_ primer (Promega, Madison, WI) according to the manufacturer’s instructions. Quantitative RT-PCR was performed by using the THUNDERBIRD SYBR quantitative PCR mix (TOYOBO Co. Ltd., Osaka, Japan). The values were standardized using *β*-*actin* (*Actb*). The primer sets used were as follows: 5′-AGCGCTTCCGGGACCTCGTA-3′ and 5-CCCGCTCTTTGCAACGCTCT-3′ for *Dhh*, 5′-CCGACCCAGATTGCCCTGCC-3′ and 5-CAGGGCGTGAGCGCTGACAAG-3′ for *Ptch1*, 5′-CAAACCCTGAGACCACAATGG-3′ and 5-TGATGCCCACATAGCCTTCAT-3′ for *Pdgfrα* [38], 5′- ATTGCTCCCCACCTCCTGGCTATG-3′ and 5- GGTCATGATGGGGCTTCTTGGGGA-3′ for *Insl3* (Insulin like 3), 5′-GGGCCTCCGAAACCATGAAC-3′ and 5′-TGAACTTGATCACTTCATGGGACT-3′ for *Vegfa* (vascular endothelial growth factor A), and 5′-AGGGTGTGATGGTGGGAATGG-3′ and 5-TGGCTGGGGTGTTGAAGGTCT-3′ for *Actb* [38].

### Triple Staining for ARX, 3β-HSD and AD4BP/SF-1

Frozen sections of ICR testes at E13.0 were immunostained for ARX, 3β-HSD, and AD4BP/SF-1 as described above. For primary antibodies, rabbit antibody against ARX and rat antibody against AD4BP/SF-1 were used. For 3β-HSD staining, protein A-purified anti-3β-HSD antibody was labeled directly with Alexa Fluor® 488 using Alexa Fluor® 488 Protein Labeling Kit (Molecular Probes, Eugene, OR). Cy3-labeled anti-rabbit antibody and Cy5-labeled anti-rat antibody (Jackson ImmunoResearch) were used as the secondary antibodies. Observation was performed by confocal microscopy. The number of 3β-HSD-positive cells, and ARX and 3β-HSD double-positive cells were counted with 4 to 7 pictures for each gonad (n = 3).

## Results

### Expression of ARX in Mouse Fetal Gonads

**Figure 1:**
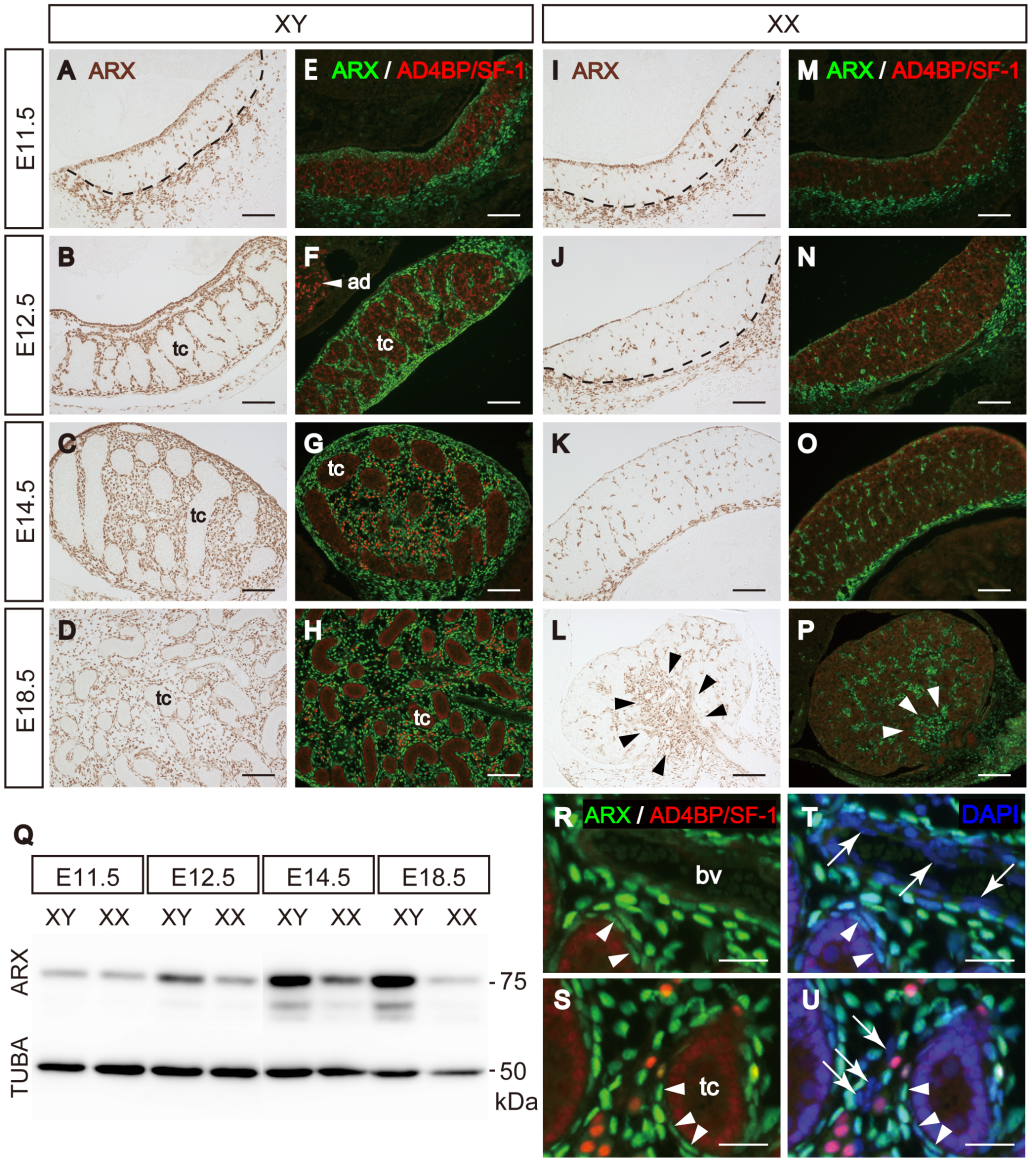
Expression of ARX in interstitial cells of fetal gonads. Expression of ARX was examined by immunohistochemistry using an anti-ARX antibody. Wild type male (XY) (A–D) and female (XX) (I–L) gonads of mouse fetuses at E11.5, E12.5, E14.5, and E18.5 were tested. Double immunofluorescent staining for ARX (green) and AD4BP/SF-1 (red) was performed with male (E–H) and female (M-P) gonads at the same stages. Dashed lines indicate the gonad-mesonephros border. Scale bars = 100 µm. Whole gonadal extracts (5 µg) prepared from mouse fetuses of both sexes at E11.5, E12.5, E13.5, E14.5, and E18.5 were subjected to western blot analysis using anti-ARX and anti-α-tubulin antibodies (Q). The location of 50 and 75 kDa protein markers are indicated. Arrowheads in L and P indicate middle part of ovary and mesovarium, respectively. Magnified views of E18.5 testis are shown (R-U). Sections are counterstained by DAPI (blue, T and U). Arrowheads in R-U indicate ARX-positive peritubular myoid cells and arrows in T and U indicate ARX-negative and DAPI-positive (blue) endothelial and unknown interstitial cells, respectively. Scale bars = 25 µm. ad, adrenal; tc, testis cord; bv, blood vessel.

We previously reported that fetal Leydig cell differentiation is largely affected in *Arx* KO testes [14]. In the present study, we examined expression of ARX in male and female mouse fetal gonads during development (Figure 1A–H for male and I-P for female). In sexually indifferent gonads at E11.5 (Figure 1A, 1E, 1I, and 1M), ARX was expressed in the coelomic epithelial cells and cells underneath the epithelium (epithelial domain). In addition, the expression was observed in the mesonephric cells and a few gonadal cells near the gonad-mesonephros border (gonad-mespnephros domain). Only a few ARX-positive cells were found to be scattered throughout the gonads. This expression was similar between the two sexes (Figure 1A, 1E, 1I and 1M). In differentiating male gonads at E12.5 (Figure 1B and 1F), ARX was expressed in the epithelial cells and interstitial cells but not in cells within the testis cords. This expression persisted during the fetal days examined (Figure 1B–D and 1F–H). In the female gonads at E12.5 and E14.5, ARX was expressed in certain mesenchymal and epithelial cells (Figure 1J, 1N, 1K, and 1O). In addition, many ARX-positive cells were localized at the border between the ovary and mesonephros. In the ovary at E18.5, these cells seemed to migrate into the middle part of the ovary (arrowheads in Figure 1L). ARX-positive cells were also seen in rete ovarii in the mesovarium (arrowheads in Figure 1P). The amount of ARX protein in the developing gonads was examined with immunoblotting (Figure 1Q). The amounts of ARX protein were low at E11.5 in both sexes, and thereafter increased in the testes but remained at low level in the ovaries, correlating well with the observations above.

To characterize ARX expressing cells, double immunostaining for ARX and AD4BP/SF-1 was performed (Figure 1E–H and 1R-U for male, and 1M-P for female). At E11.5, many AD4BP/SF-1-positive cells were present in the mesenchymal compartments of the gonads of both sexes. Many of these AD4BP/SF-1-positive cells were not positive for ARX (Figure 1E and 1M). As mentioned above, after testis cords were formed in the male gonad, most interstitial cells were ARX-positive at E12.5, E14.5, and E18.5 (Figure 1F–H). During these stages, fetal Leydig cells, recognized as AD4BP/SF-1-strong positive cells, increased in the interstitial space and were unlikely to be positive for ARX. However, as will be described below, some but not all cells showing a weak signal for AD4BP/SF-1 seemed to be weakly positive for ARX. Similar to the testis, expression of AD4BP/SF-1 and ARX was basically exclusive in the ovaries (Figure 1M–P).

In addition to fetal Leydig cells, other cell types such as peritubular myoid cells surrounding testis cords, vascular endothelial cells, and uncharacterized cells are present in the interstitial space [39]. ARX was expressed in the peritubular myoid cells (arrowheads in Figure 1R-U) but not in vascular endothelial cells (arrows in Figure 1T). In addition, ARX was expressed in many, though not all, uncharacterized cells (arrows in Figure 1U).

### Testicular Development Affected in Arx KO Mice

**Figure 2:**
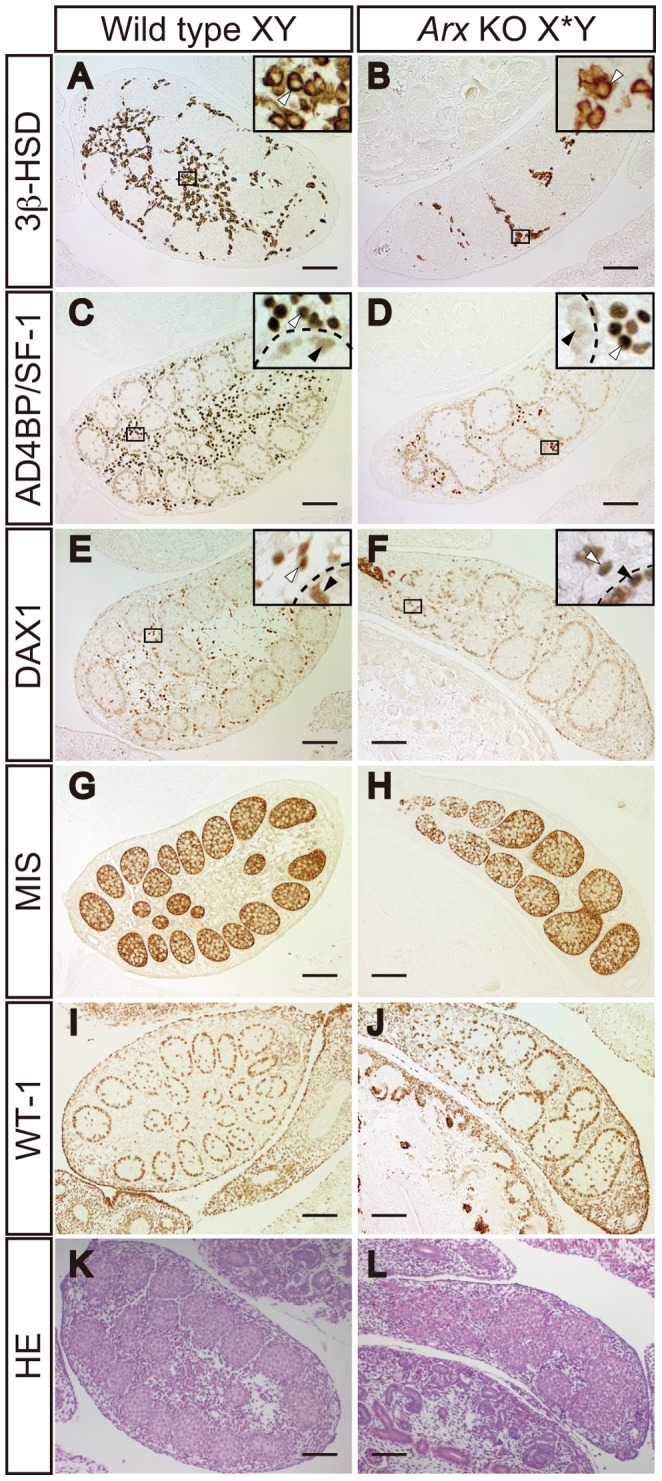
Expression of Sertoli and Leydig cell marker proteins in *Arx* KO testes. Expression of Leydig cell markers, 3β-HSD (A and B), AD4BP/SF-1 (strong signals outside of the testis cords) (C and D), and DAX-1 (strong signals outside of the testis cords) (E and F), and Sertoli cell markers, AD4BP/SF-1 (weak signals in testis cords) (C and D), DAX-1 (weak signals in testis cords) (E and F), MIS (G and H), and WT-1 (I and J) in testes of wild type (Wild type XY) and *Arx* KO (*Arx* KO X*Y) mice at E14.5 was investigated by immunohistochemistry. HE staining was also performed for the specimens (K and L). Enclosed areas in A–F are enlarged at the top right in each panel. White and black arrowheads indicate Leydig cells and Sertoli cells, respectively. Dashed lines indicate testis cord. Scale bars = 100 µm.

The effects of the gene disruption on Leydig and Sertoli cell differentiation were examined in terms of marker gene expression. In wild type testes, many cells were positive for the Leydig cell marker 3β-HSD at E14.5, whereas in the *Arx* KO testes the number of 3β-HSD-positive cells was apparently decreased (Figure 2A and 2B). AD4BP/SF-1 and DAX-1 were expressed strongly in fetal Leydig cells (Figure 2C and 2E, white arrowheads) and weakly in Sertoli cells (Figure 2C and 2E, black arrowheads). Consistent with the 3β-HSD staining, the number of fetal Leydig cells, which were strong positive for AD4BP/SF-1 and DAX-1, was decreased in the KO testes (Figure 2C–F, white arrowheads). In contrast, the expression of the MIS and WT-1 in Sertoli cells of wild type appeared to be unaltered in the KO testes (Figure 2G, 2H, 2I and 2J). Similarly, the weak expression of AD4BP/SF-1 and DAX-1 in Sertoli cells was unaltered in the KO testes (Figure 2C–F, black arrowheads).

**Figure 3:**
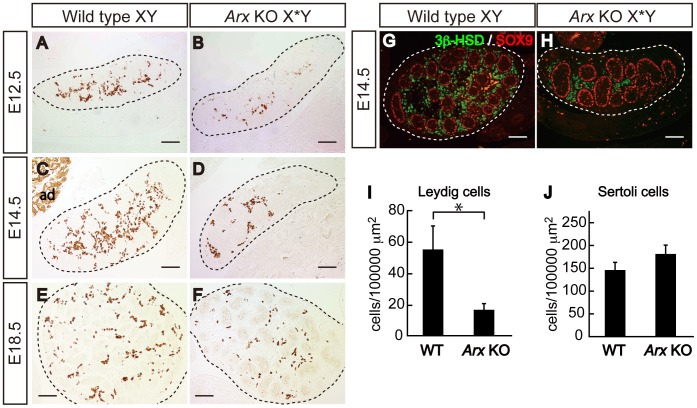
Reduction in fetal Leydig cells in *Arx* KO testes. Expression of Leydig cell marker 3β-HSD was analyzed in wild type (Wild type XY) (A, C and E) and *Arx* KO (*Arx* KO X*Y) (B, D and F) gonads at E12.5, E14.5, and E18.5. Double immunofluorescent staining for 3β-HSD (green) and SOX9 (red) was performed with wild type (G) and *Arx* KO (H) gonads at E14.5. Dashed lines indicate the gonads. The numbers of Leydig and Sertoli cells were counted using the sections prepared from E14.5 testes and are shown per unit area (I and J). The data are shown as the mean ± SD. * P<0.05. Scale bars = 100 µm.

We examined chronologically the defect in Leydig cell differentiation from E12.5 to E18.5 (Figure 3A–F). Although the number of 3β-HSD-positive fetal Leydig cells increased with increasing fetal age in both wild type and KO, the numbers were less in the KO at all stages examined than those in wild type (Figure 3A–F). In fact, the number of fetal Leydig cells per unit area of the KO testis was approximately 30% of that in wild type (Figure 3I). In contrast, SOX9-positive Sertoli cells did not appear to be reduced (Figure 3G and 3H). Consistent with it, the number of Sertoli cells per unit area was similar in both the KO and wild type (Figure 3J).

### Proliferation of Fetal Leydig Cells

**Figure 4:**
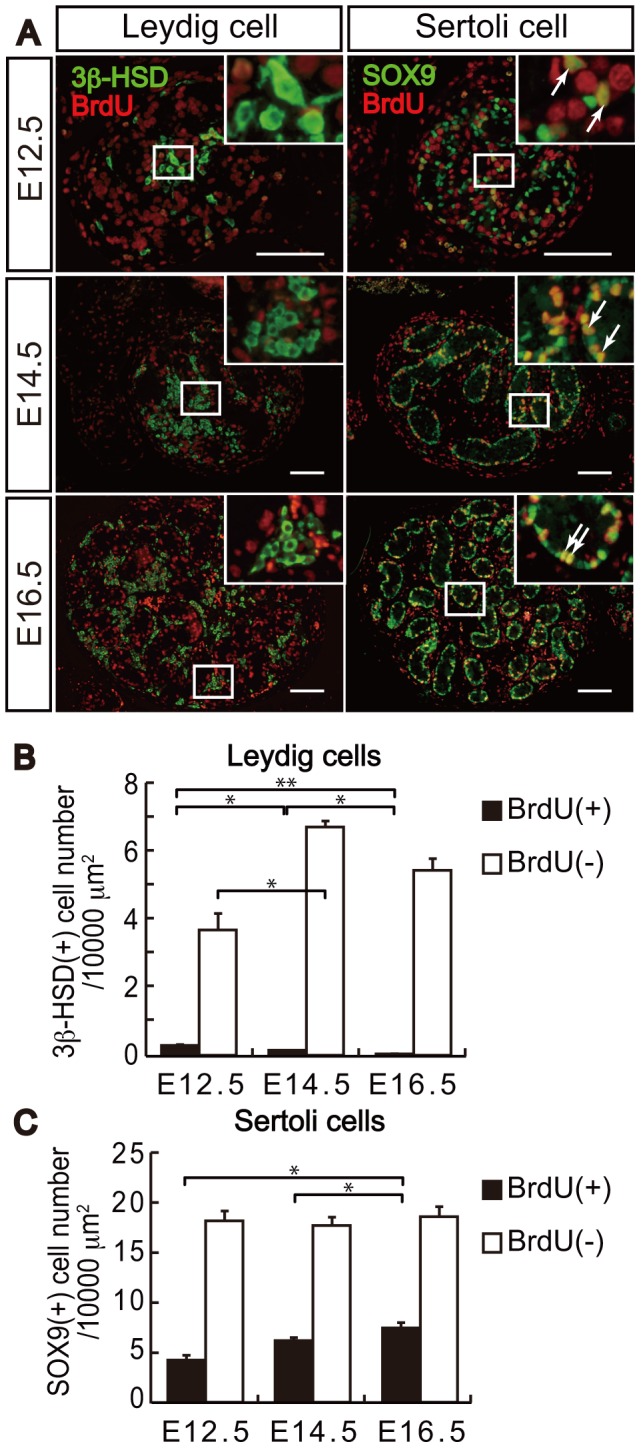
Suppressed proliferation in fetal Leydig cells. Cell proliferation was evaluated by BrdU incorporation studies with three fetal testes. BrdU labeled fetal testes at E12.5, E14.5 and E16.5 were sectioned and the sections were immunostained with antibodies for 3β-HSD (green) and BrdU (red) (left panels in A; Leydig cell), and with SOX9 (green) and BrdU (red) (right panel in A; Sertoli cell). Enclosed areas are enlarged at the top right in each panel. Arrows indicate cells double-positive for SOX9 and BrdU. Scale bars = 100 µm. Numbers of 3β-HSD and BrdU double-positive cells (closed bars) and 3β-HSD single-positive cells (open bars) (B), and numbers of SOX9 and BrdU double-positive cells (closed bars) and SOX9 single-positive cells (open bars) (C) were counted (n = 3). The numbers of these cells per unit area are plotted. The data are indicated as the mean ± SD. *, P<0.05, **, P<0.01.

In order to elucidate why *Arx* gene disruption led to a decrease in fetal Leydig cells, we investigated whether this decrease was due to a decreased rate of cell proliferation. We initially examined cell proliferation in wild type testes. After fetuses in utero were labeled with BrdU at E12.5, E14.5, and E16.5, proliferation of fetal Leydig and Sertoli cells was evaluated (Figure 4). At E12.5, many SOX9-positive Sertoli cells and SOX9-negative germ cells in the testis cords were labeled with BrdU. Similarly, many cells in the interstitial space were BrdU-positive. However, there were only a small number of BrdU and 3β-HSD double-positive cells. Thereafter, male germ cells are known to stop proliferation [5]. In fact, BrdU-positive and SOX9-negative germ cells in the testis cord were only minimally detected at E14.5 and E16.5. In contrast, Sertoli cells were still proliferating (arrows in Figure 4A). Only a small population of 3β-HSD-positive Leydig cells proliferated at E14.5 and E16.5. The proliferating Sertoli and Leydig cells were counted and shown as the number of cells per unit area in Figure 4B and 4C. Approximately 20–30% of Sertoli cells were labeled with BrdU during the stages examined. By contrast, less than 6.5% of fetal Leydig cells were labeled with BrdU at E12.5, and thereafter the labeling rate was decreased, indicating that fetal Leydig cells undergo minimal proliferation during the fetal ages. It is well known that in these stages fetal Leydig cells rapidly increase in number. Accordingly, it is possible that this increase should be achieved by vigorous differentiation from progenitor cells to mature fetal Leydig cells. Therefore, decrease of fetal Leydig cells in *Arx* KO testes is possibly due to affected differentiation of fetal Leydig cells. However, we cannot exclude a possibility that proliferation of progenitor fetal Leydig cells is affected by *Arx* gene disruption at stage earlier than E12.5.

Since increased apoptotic cell death was thought to be another possibility, we examined whether apoptosis was induced in *Arx* KO Leydig cells by immunostaining of 3β-HSD and apoptotic marker, active-type caspase 3. However, we could not detect apoptotic fetal Leydig cells in the gonads of *Arx* KO as well as wild type. Considering these results, the reduction of fetal Leydig cells in *Arx* KO testes is unlikely to be due to a decreased rate of cell proliferation or increased rate of apoptosis of Leydig cells.

### Defects other than those in Fetal Leydig Cells

**Figure 5:**
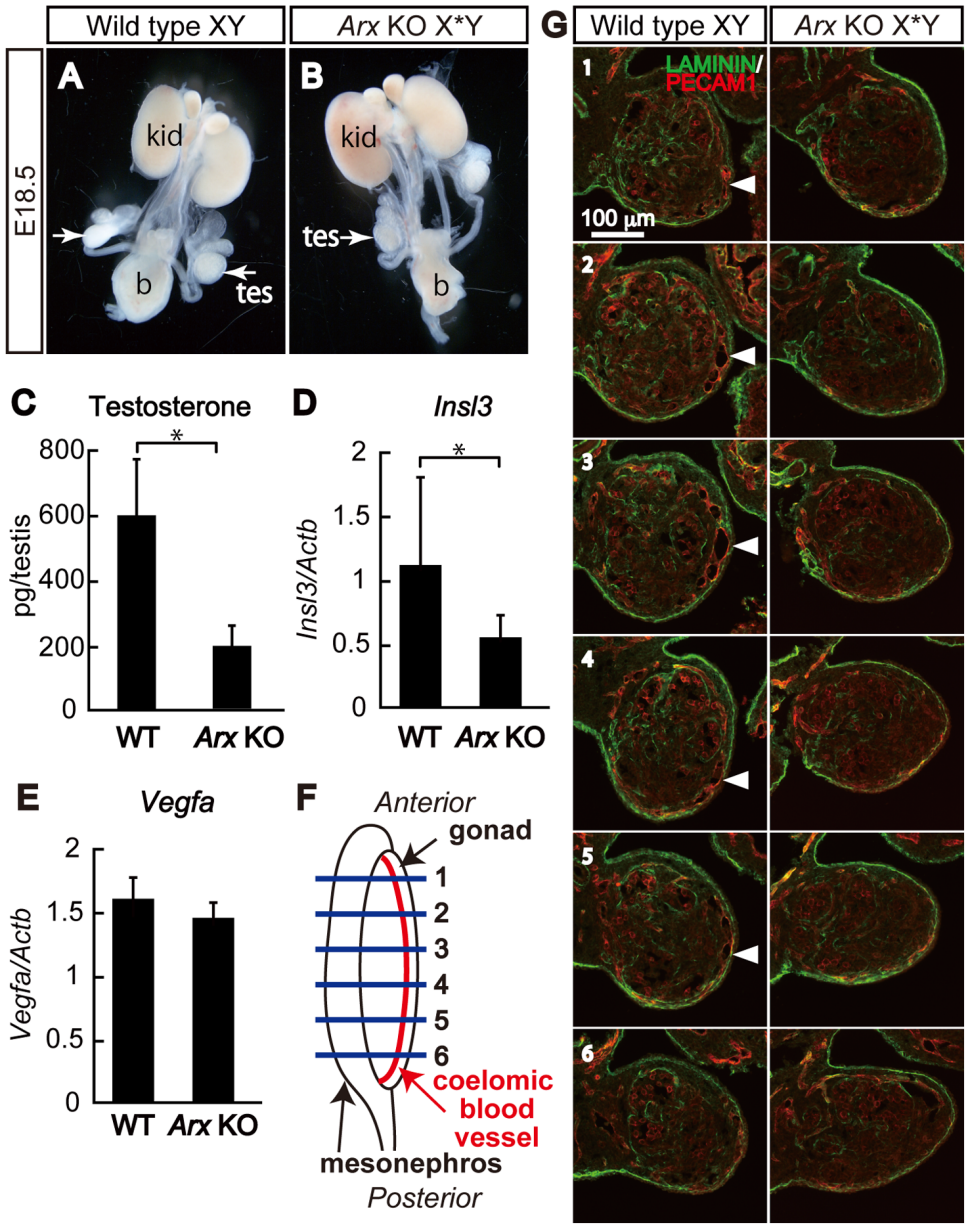
Structural abnormalities induced in *Arx* KO mouse. Urogenital systems of wild type (Wild type XY) (A) and *Arx* KO (*Arx* KO X*Y) (B) male mice at E18.5 are shown. Arrows indicate the testes. tes, testis; b, bladder; kid, kidney. Intratesticular testosterone levels of wild type (Wild type XY) and *Arx* KO (*Arx* KO X*Y) testes were measured at E18.5 (C). The data are indicated as the mean ± SD. *, P<0.05. Expression of *Insl3* and *Vegfa* in wild type (Wild type XY) and *Arx* KO (*Arx* KO X*Y) testes at E12.5 was determined by quantitative RT-PCR (D and E). The data were standardized using *β-actin* (Actb) and shown as the mean ± SD. *, P<0.05. Locations of the sections of the gonad are schematically shown with horizontal lines with numerals, 1 to 6 (F). The coelomic blood vessel is indicated with red line. Double immunofluorescent staining for LAMININ (green) and PECAM (red) was performed with the serial sections (1 to 6, corresponding to the numerals in (E)) with 100 µm interval of wild type and *Arx* KO mouse testes at E12.5 (G). Arrowheads indicate the coelomic blood vessel in wild type, while the corresponding structure could not be observed at this stage in the KO testis. Scale bars = 100 µm.

It has previously been reported that *Arx* KO mice develop smaller testes and hypoplastic seminal vesicles [14]. In the present study, we found that the testes were frequently undescended in the KO mice (Figure 5A and 5B). Considering that antiandrogen treatment affects seminal vesicle development and testicular descent [40], and that this treatment results in smaller testes development in fetal age [41], the defects seen in *Arx* KO mice are thought to be dependent, at least in part, on the decrease of testosterone production caused by the decrease of fetal Leydig cells. In fact, intratesticular testosterone level was significantly low in the KO testes, compared with wild type testes (Figure 5C). INSL3 has been known to be another factor to regulate testicular descent [42], [43]. Then, the expression of *Insl3* in the KO testes was examined by RT-PCR. Expectedly, *Insl3* was decreased to nearly one-half of wild type (Figure 5D).

Another obvious defect was the delayed development of a coelomic blood vessel in *Arx* KO testes. Usually, a large coelomic blood vessel can be detected at the surface of the testis at E12.5, whereas such a prominent vessel is not seen in the ovary. As shown in the serial sections of wild type and *Arx* KO testes (Figure 5F and 5G), the coelomic blood vessel was very thin, if any, or not present in the *Arx* KO testis at E12.5. Interestingly, however, it was formed at a later stage (E13.5, data not shown). Since the formation of this blood vessel has been shown to be independent of testosterone action [41], *Arx* may be involved in a testosterone independent pathway as well as a testosterone dependent pathway during testis development. Since inhibition of VEGF-A prevents coelomic blood vessel formation [44], we examined the expression of *Vegfa* in KO testes at the same stages. However, the expression of *Vegfa* was not affected in the KO gonads at E12.5 (Figure 5E).

### Expression of Genes Required for Fetal Leydig Cell Differentiation

**Figure 6:**
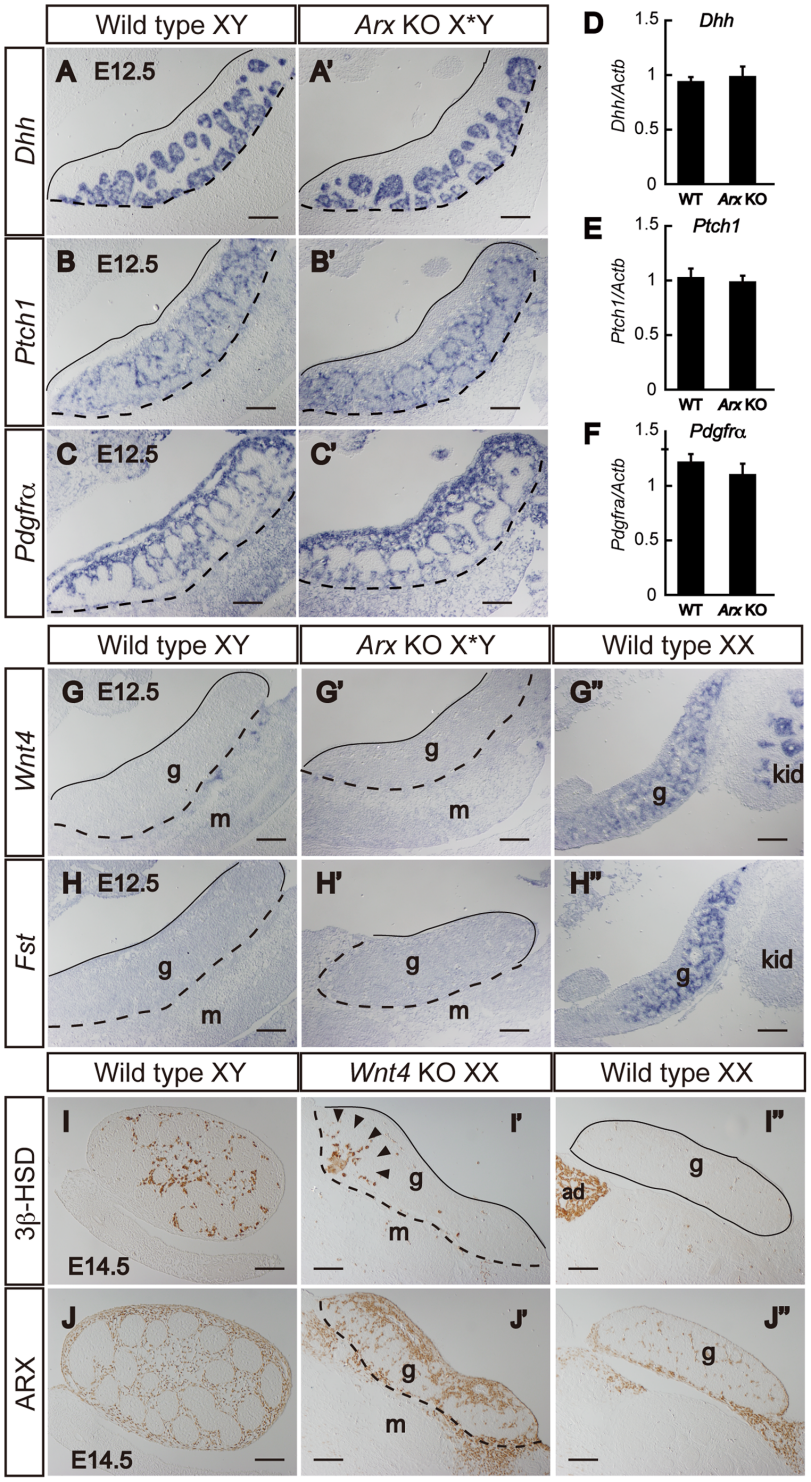
Expression of gonadal marker genes in *Arx* KO and *Wnt4* KO gonads. Expression of *Dhh* (A and A’), *Ptch1* (B and B’), *Pdgfrα* (C and C’), *Wnt4* (G-G’’) and *Fst* (H-H’’) in wild type male (Wild type XY), *Arx* KO male (*Arx* KO X*Y) and wild type female (Wild type XX) gonads was examined by *in situ* hybridization and quantitative RT-PCR (D-F). Quantitative RT-PCR (D–F) was performed at E12.5. The data were standardized using *β-actin* (Actb) and shown as the mean ± SD. Expression of 3β-HSD and ARX in wild type male (Wild type XY), *Wnt4* KO female (*Wnt4* KO XX) and wild type female (Wild type XX) gonads was also examined by immunohistochemistry (I–I’’ and J–J’’). Lines and dashed lines indicate the gonadal epithelium and gonad-mesonephros border. Scale bars = 100 µm. g, gonad; m, mesonephros; kid, kidney; ad, adrenal.

The reduction of Leydig cell numbers in *Arx* KO fetal testes suggested that expression of genes required for fetal Leydig cell differentiation is affected in the KO testes. Studies with *Dhh* [7] and *Pdgfrα* [9] KO mice have demonstrated that these genes are required for fetal Leydig cell differentiation. Therefore, we examined whether the expression of these genes is downregulated in *Arx* KO testes. *Dhh* and its receptor *Ptch1* were expressed in Sertoli and interstitial cells, respectively, in wild type testes (Fig. 6A and 6B), and theses expression patterns were unaffected in the *Arx* KO testes (Figure 6A’ and 6B’). *Pdgfrα* was expressed in interstitial cells of wild type testes and the expression pattern was also unaffected in the KO testes (Figure 6C and 6C’). Consistent with these results, the amounts of the mRNA for *Dhh*, *Ptch1*, and *Pdgfrα* were not decreased in the KO testes (Figure 6D, 6E, and 6F). Even though any clear difference in these gene expressions was not observed, it is interesting to note that the region between the epithelium and testis cords (epithelial domain) seemed to be expanded in the KO (compare Figure 6A with 6A’, 6B with 6B’, and 6C with 6C’).

*Wnt4* is expressed more in the fetal ovary than in the testis, and steroidogenic (Leydig) cells develop ectopically in *Wnt4* KO fetal ovaries, suggesting that the expression of *Wnt4* suppresses steroidogenic (Leydig) cell differentiation in the ovary [11]. Alternatively, the steroidogenic cells seen in the *Wnt4* KO ovary have been discussed to be derived from mis-migrated adrenocortical cells [45], [46]. Therefore, *Wnt4* expression was assumed to be affected in *Arx* KO testes. Unexpectedly, however, the expression was not affected in the KO (Figure 6G–G’’). The expression of *Fst* is regulated positively by *Wnt4* and thus enriched in fetal ovaries [47]. Similar to *Wnt4*, the expression of *Fst* was not altered in the KO (Figure 6H–H’’). We next examined the possibility that the expression of *Arx* is affected in *Wnt4* KO mice. As previously reported [11], 3β-HSD-positive steroidogenic cells, which are normally absent from wild type ovaries, were differentiated in the *Wnt4* KO ovaries (Figure 6I–I’’). Similarly, the expression of ARX was induced in the KO ovary (Figure 6J–J’’), indicating that *Wnt4* suppresses the *Arx* gene expression in the ovary.

### Presence of Interstitial Cells Showing Overlapped Expression of ARX and Leydig Cell Markers

**Figure 7:**
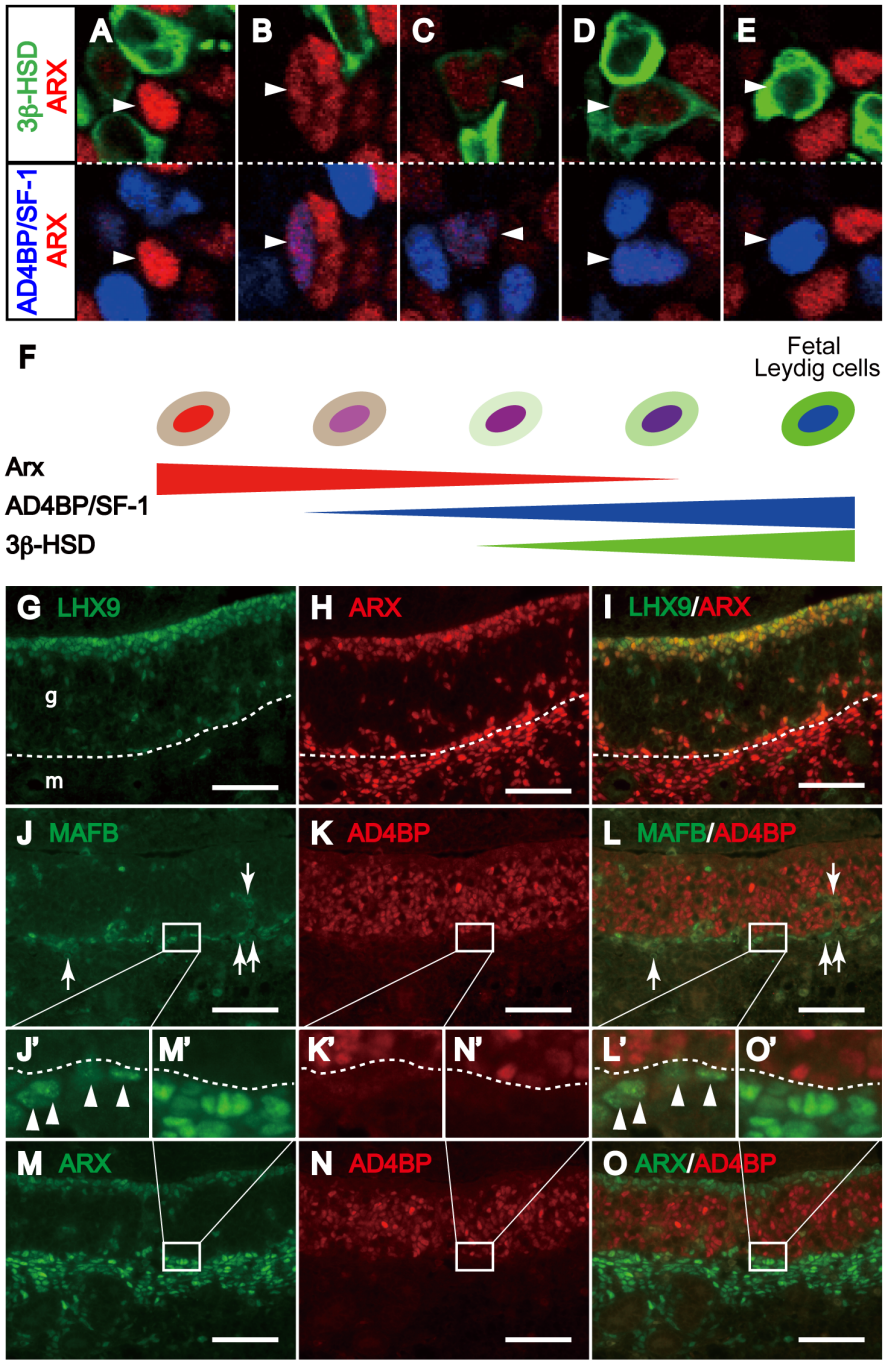
Expression of ARX, 3β-HSD, LHX9, and AD4BP/SF-1 in interstitial cells of fetal testes. Expression of ARX (red), AD4BP/SF-1 (blue), and 3β-HSD (green) in fetal testis at E13.0 was examined. Merged images of ARX and 3β-HSD (upper panels of A–E), and ARX and AD4BP/SF-1 (lower panels of A–E) are shown. The arrowhead in A indicates an ARX-strongly positive, AD4BP/SF-1-negative, and 3β-HSD-negative cell. The arrowhead in B indicates an ARX-weakly positive, AD4BP/SF-1-weakly positive, and 3β-HSD-negative cell. The arrowhead in C indicates an ARX-weakly positive, AD4BP/SF-1-weakly positive, and 3β-HSD-weakly positive cell. The arrowhead in D indicates an ARX-weakly positive, AD4BP/SF-1-strongly positive, and 3β-HSD-modestly positive cell. The arrowhead in E indicates an ARX-negative, AD4BP/SF-1-strongly positive, and 3β-HSD-strongly positive cell. Cells showing expression of the marker proteins above are illustrated (F). Expression of LHX9 (green) and ARX (red) in E11.5 male gonads was examined (G-I). Dashed lines in G-I indicate the gonad-mesonephros border. Expressions of MAFB (green) and AD4BP/SF-1 (red), and ARX (green) and AD4BP/SF-1 (red) in E11.5 male gonads were examined on consecutive sections (J–O, and J’–O’ for enlarged views). Arrows in J and L indicate autofluorescence of blood cells. Dashed lines in J’–O’ indicate border between MAFB or ARX-positive cells and AD4BP/SF-1-positive cells. Arrowheads in J’ and L’ indicate MAFB-positive cells just beneath AD4BP/SF-1-positive cells. Scale bars = 100 µm. g, gonad; m, mesonephros.

The results described above indicated that *Arx* is not expressed in fetal Leydig cells. Nevertheless, *Arx* gene disruption affected fetal Leydig cell differentiation. This seemingly inconsistent observation raised two possibilities concerning the expression and function of *Arx*. The first is that *Arx* is expressed in cells other than those of Leydig cell lineage and thus stimulates progenitor cells to differentiate into fetal Leydig cells, while the second is that *Arx* is expressed in the progenitors of fetal Leydig cells and functions at the progenitor stage. In order to determine whether ARX expressing cells, if not all, are the progenitors of fetal Leydig cells, we analyzed the expression of ARX and Leydig cell markers, AD4BP/SF-1 and 3β-HSD. Similar to the results in Figure 1, typical ARX-strongly positive cells were negative for both AD4BP/SF-1 and 3β-HSD (arrowheads in Figure 7A), while typical AD4BP/SF-1 and 3β-HSD -strongly positive fetal Leydig cells were negative for ARX (arrowheads in Figure 7E). In addition to these cells, there were a small number of cells showing atypical expression of these marker proteins. As shown in Figure 7B, there were cells weakly positive both for ARX and AD4BP/SF-1, and negative for 3β-HSD. In addition, there were cells weakly positive both for ARX and 3β-HSD (Figure 7C and 7D, arrowheads). The percentage of ARX and 3β-HSD double-positive cells to total 3β-HSD-positive cells was approximately 5%. As summarized in Figure 7F, it was assumed that these cells might be transitional cells from ARX single-positive progenitor cells to AD4BP/SF-1 and 3β-HSD double-positive fetal Leydig cells.

*Lhx9* has been shown to be expressed in undifferentiated cell population in the testicular interstitium [48]. Then, the expression of LHX9 in E11.5 testes was examined. As described above, ARX was expressed in the cells of two domains, epithelial domain and gonad-mesonephros domain (Figure 1A, 1E, and Figure 7H), while LHX9 was expressed predominantly in the epithelial domain (Figure 7G). These two factors seemed to be co-expressed nearly all cells in the epithelial domain although the mutual expression levels were different among the cells (Figure 7I). *Mafb* was reported to be expressed in precursors of Leydig cells [48]. Then, we performed immunostaining of MAFB (Figure 7J–L and 7J’–L’) and ARX (Figure 7M–O and 7M’–O’) on consecutive sections at E11.5 since the antibodies for them were both raised in rabbits. As the control, AD4BP/SF-1 was immunostained. A few MAFB-positive cells were found in the gonadal mesenchymal compartment, while many MAFB-positive cells were in the gonad-mesonephros domain (arrowhead in Figure 7J’ and 7L’) where ARX was widely expressed (Figure 7M’ and 7O’). Considering that the expressions of MAFB and Ad4BP/SF-1 were basically exclusive as those of ARX and AD4BP/SF-1 (Figure 7J’ and 7K’, and 7M’ and 7N’, respectively), MAFB-positive cells seemed to be overlapped with ARX-positive cells in the gonad-mesonephros domain (Figure 7J’–O’).

## Discussion

### Progenitors of Fetal Leydig Cells

During early testis development, the testis is divided into two compartments, the testis cord and interstitial space. Two cell populations have been proposed as the possible source of interstitial cells. One is the cells distributed at the gonad-mesonephros border and the other is the coelomic epithelial cells. The former were shown to migrate internally into the testis and thereafter differentiate into peritubular myoid cells and endothelial cells [49]–[52]. A recent study investigating Maf transcription factors, *Mafb* and *c-Maf*, found that in the gonads at sexually indifferent stages the former is expressed in the coelomic epithelial cells and cells underneath the epithelium (epithelial domain), and the cells around gonad-mesonephros border (gonad-mesonephros domain), while the latter is expressed in the gonad-mesonephros domain. Moreover, some of the MAFB and C-MAF expressing cells differentiate into interstitial cells including fetal Leydig cells after migration into the interstitial space [48]. Our present study showed that ARX is expressed in the cells possibly corresponding to the two cell populations where MAFB and C-MAF are expressed. In addition to these *Mafs*, *Lhx9* has also been discussed to be expressed in undifferentiated gonadal cells. Our study demonstrated that LHX9 and ARX are co-expressed in nearly all cells in the epithelial domain of the gonad. Together with the distribution of MAFs [48], the overlapped distribution between LHX9 and ARX may suggest that ARX is expressed in the progenitors of fetal Leydig cells and regulate differentiation of the progenitor cells to mature fetal Leydig cells.

### Fetal Leydig Cells Affected at the Progenitor Stage in Arx KO Mice

In the present study, we showed that the numbers of fetal Leydig cells in *Arx* KO mouse testes are less than one third of those found in wild type testes. This decrease may be related to the possibility that proliferation of fetal Leydig cells is affected in *Arx* KO testes. With respect to proliferation of fetal Leydig cells of wild type testes, a ^3^H-thymidine incorporation studies demonstrated that fetal Leydig cells rarely proliferate whereas Sertoli cells vigorously proliferate in rat [53], [54] and mouse [55]. However, mouse study was performed from E14.5 onward, and it was unclear whether at earlier stages fetal Leydig cells can proliferate. We examined this with mouse fetuses at E12.5, E14.5, and E16.5, and demonstrated that fetal Leydig cells do not proliferate or have a very little, if any, activity for proliferation in these days. Considering the results from our study and the previous studies, we concluded that fetal Leydig cells, once matured, scarcely proliferate during the whole fetal age. We therefore reasoned that the decrease of fetal Leydig cells in *Arx* KO is not due to a proliferation defect of mature Leydig cells, but may be due to defects induced at the progenitor stage of fetal Leydig cells.

### Possible Function of Arx during Fetal Leydig Cell Differentiation

The cells stained strongly with ARX did not appear to be stained with either of the fetal Leydig cell markers, AD4BP/SF-1 or 3β-HSD. This mutually exclusive staining may suggest that ARX functions in fetal Leydig progenitor cells and should be suppressed when the progenitor cells differentiate into fetal Leydig cells. Based on this assumption, we anticipated that it would be possible to detect cells in transition from the progenitors to mature fetal Leydig cells. We found cells that were weakly positive for ARX and AD4BP/SF-1 but negative for 3β-HSD, weakly positive for ARX, AD4BP/SF-1 and 3β-HSD, and weakly positive for ARX, strongly positive for AD4BP/SF-1 and modestly positive for 3β-HSD. These cells might be in the process of transition from progenitor cells (ARX-positive but negative for AD4BP/SF-1 and 3β-HSD) to mature fetal Leydig cells (ARX-negative and strongly positive for AD4BP/SF-1 and 3β-HSD). While the presence of these cells may support this transition, cell lineage studies would be required to determine definitely whether ARX-positive cells contain the progenitors of fetal Leydig cells. In relation to this notion, it is interesting to note that cell layer between the epithelium and testis cords became to be thicker in *Arx* KO than wild type. Given that these cells are derived from epithelial cells and migrate to the inside of the testes, migration of these cells might be affected in the KO testes. In this case, ARX is thought to regulate fetal Leydig cell differentiation at the step of migration of the progenitor cells.

The phenotypes of *Arx* KO testes indicated that this gene is implicated in the differentiation of fetal Leydig cells. However, *Arx* is unlikely to be essential for the process since fetal Leydig cells did not disappear completely from the KO testes. Moreover, gene knockout and transgenic mouse studies to date have implicated several genes in fetal Leydig cell differentiation [7], [9], [12], yet the expression of these genes was largely unaffected in *Arx* KO testis. Considering these results, multiple signals functioning complementarily and synergistically are likely to be important in the differentiation process of fetal Leydig cells.

In the present study, we examined the expression of *Arx* and defects induced in *Arx* KO fetal testes. Detailed examination of *Arx* gene expression in the fetal testes suggested that ARX may be expressed in fetal Leydig progenitor cells and thus the KO fetal Leydig cells are affected at the progenitor stage. Based on these results, we propose that ARX functions as a positive regulator for the differentiation of fetal Leydig cells through functioning at the progenitor stage.
